# Supplementary figures and images for: Exploring the changing landscape of cell-to-cell variation after CTCF knockdown via single cell RNA-seq
Source: BMC Genomics. 2019 Dec 26;20:1015. doi: 10.1186/s12864-019-6379-5 (PMC6933653; doi:10.1186/s12864-019-6379-5)

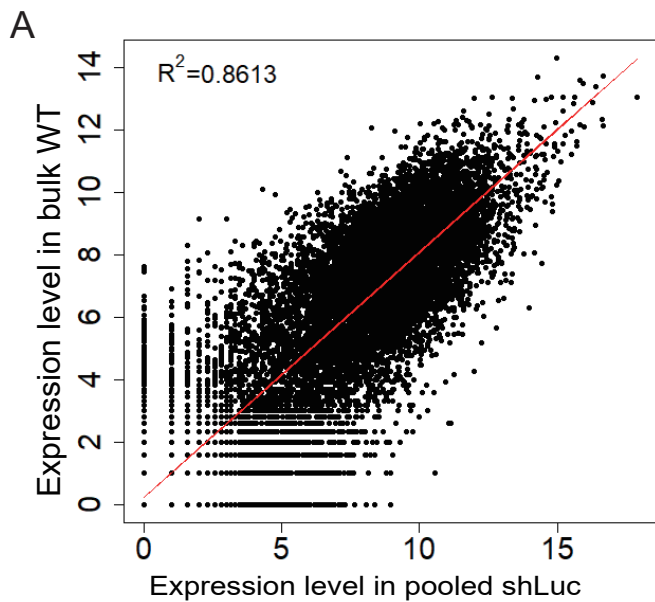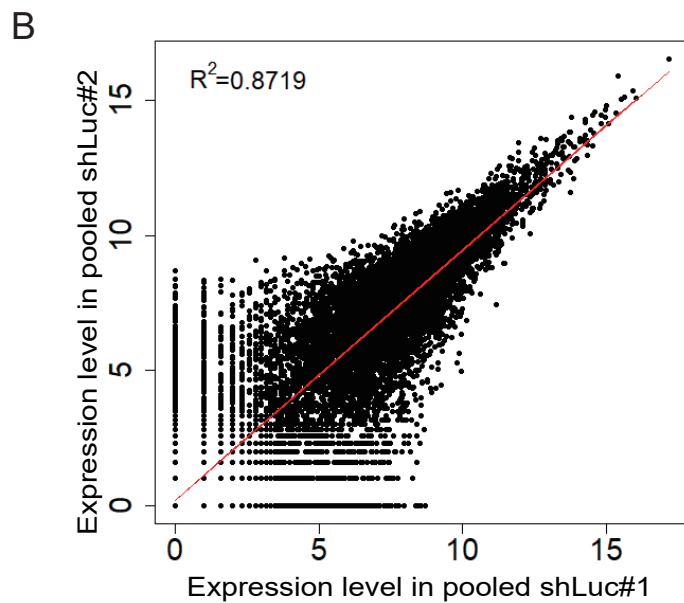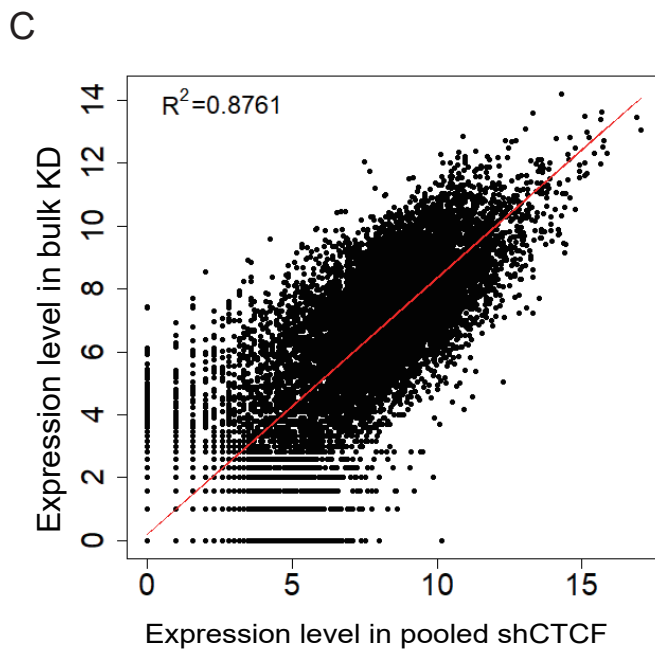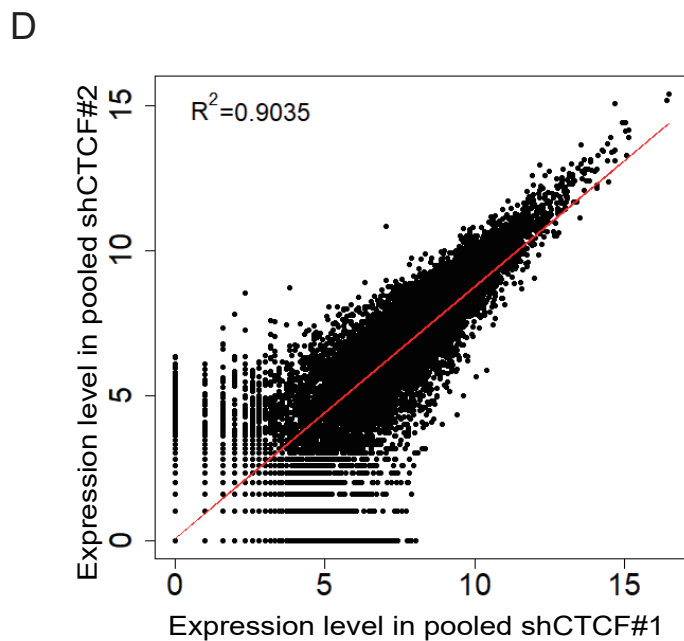

Supplement: Supplementary file 1 — Additional file 1: Figure S1. High reproducibility of single cell RNA-seq data. (A) Scatter plot showing the correlation of gene expressions between pooled single cells and bulk data in shLuc. (B) Scatter plot showing the correlation of gene expression between shLuc #1 and shLuc#2. (C) Scatter plot showing the correlation of gene expressions between pooled single cells and bulk data in CTCF-KD cells. (D) Scatter plot showing the correlation of gene expressions between shCTCF#1 and shCTCF#2. [file 12864_2019_6379_MOESM1_ESM.pdf]

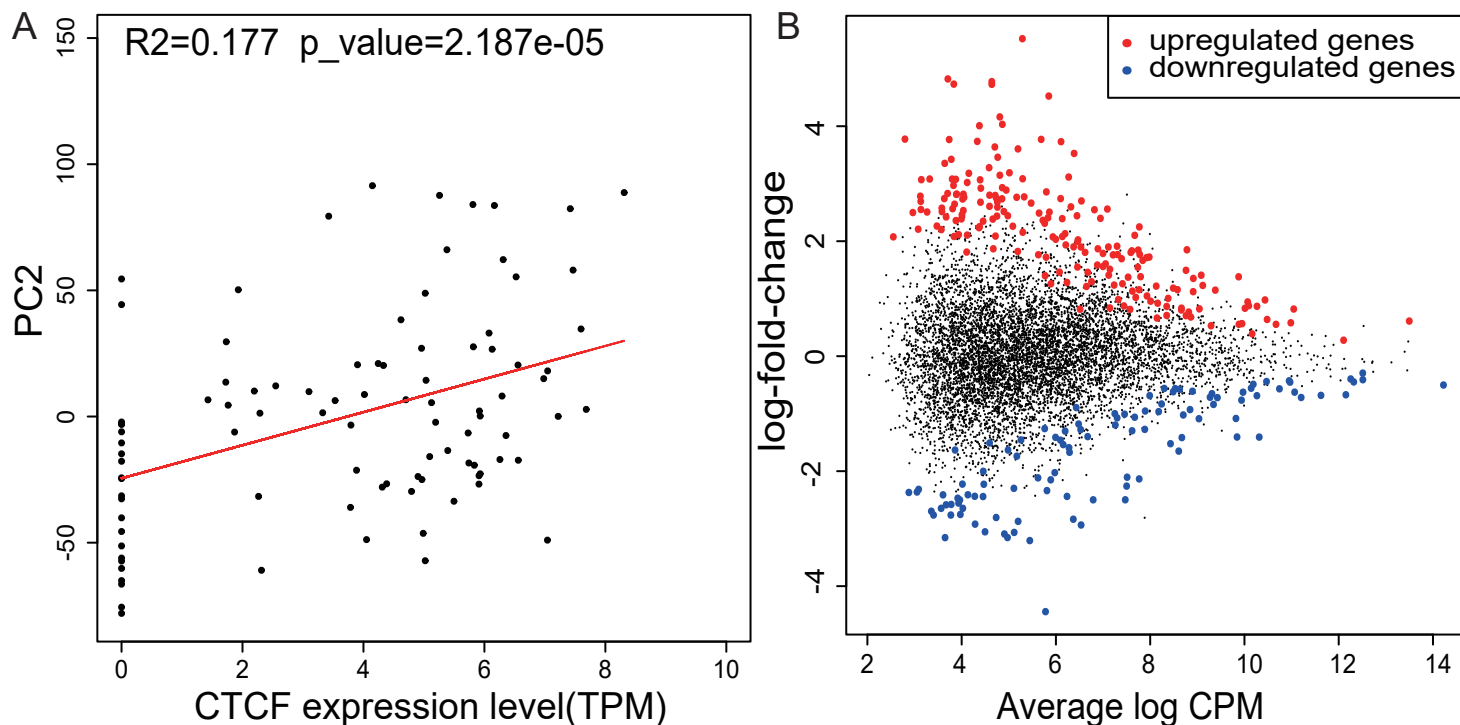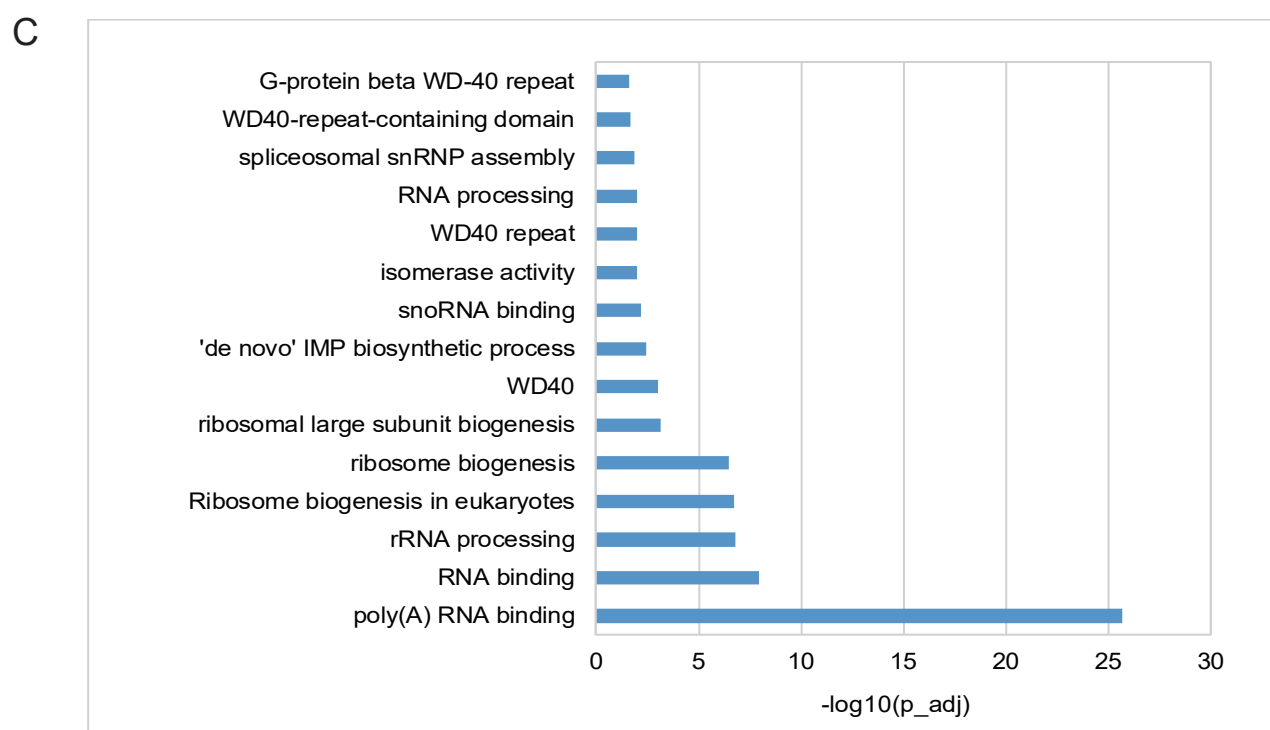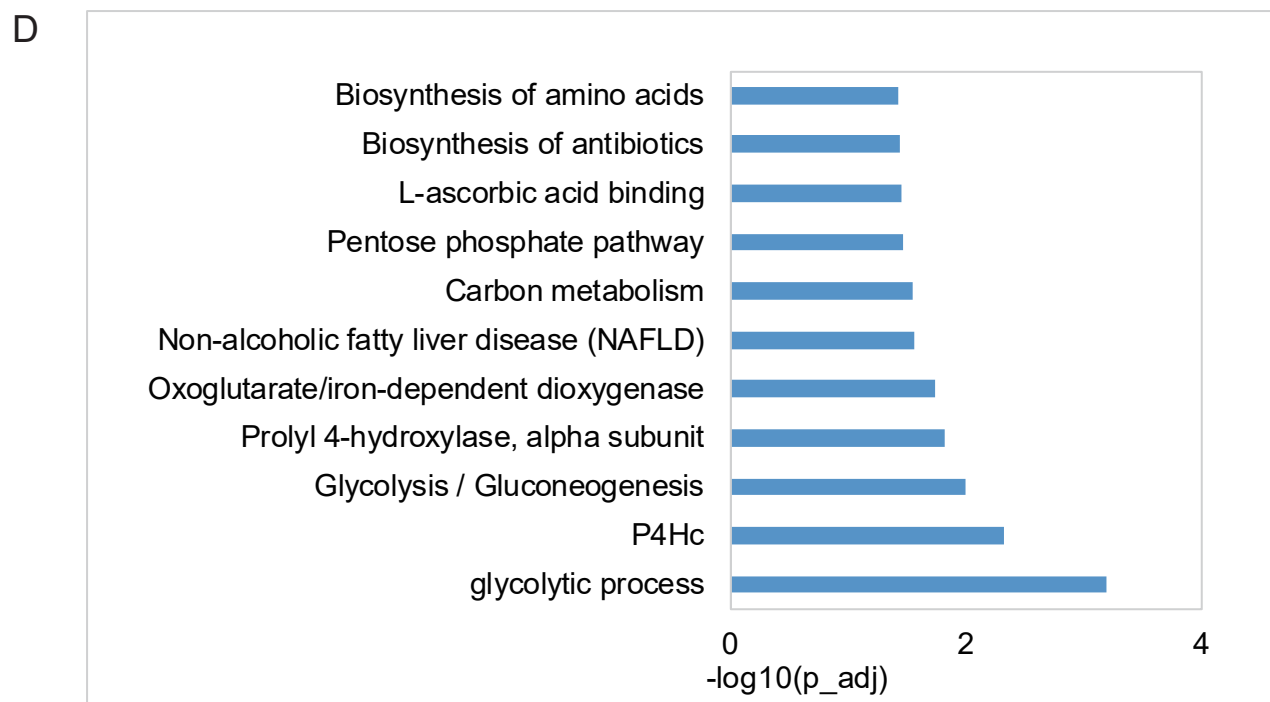

Supplement: Supplementary file 2 — Additional file 2: Figure S2. Analysis of differentially expressed genes between WT cells and CTCF KD cells. (A) The expression of CTCF is correlated with the cell coordination on PC2. (B) Differentially expressed genes were plotted in MAplot. Significantly up-regulated genes and down-regulated genes were indicated red and blue, respectively. (C) The top 15 enriched GO terms in the 195 up-regulated genes. (D) The top 15 enriched GO terms in the 107 down-regulated genes. [file 12864_2019_6379_MOESM2_ESM.pdf]

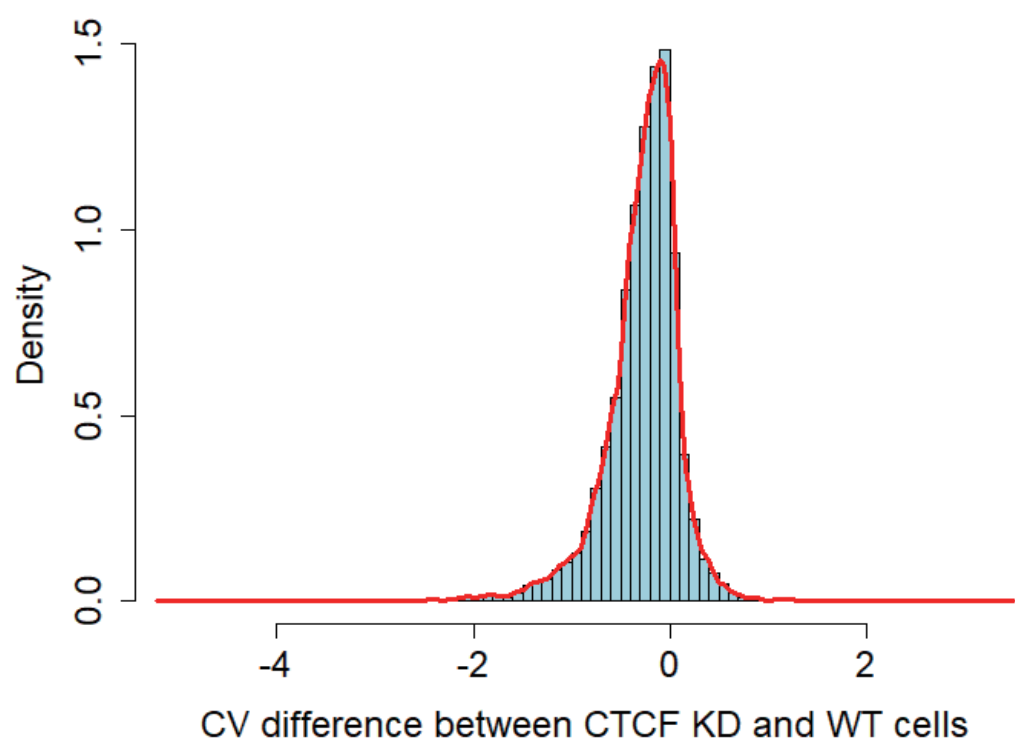

Supplement: Supplementary file 3 — Additional file 3: Figure S3. Histogram showing that CV difference of gene expression between CTCF-KD cells and WT cells followed the normal distribution. [file 12864_2019_6379_MOESM3_ESM.pdf]
